# P102: Healthcare workers’ hand contamination levels and antimicrobial efficacy of different hand hygiene methods used in a Vietnamese hospital

**DOI:** 10.1186/2047-2994-2-S1-P102

**Published:** 2013-06-20

**Authors:** S Salmon, ML McLaws, TA Truong, HV Nguyen, D Pittet

**Affiliations:** 1 UNSW Medicine, UNSW, Sydney, Australia; 2 Infection Control, Bach Mai Hospital, Ha Noi, Vietnam; 3 University of Geneva Hospitals and Faculty of Medicine, Geneva, Switzerland

## Introduction

The quality of water in Viet Nam for handwashing with soap or other disinfectant solutions is unknown. We assessed the risk for hand contamination and compared the efficacy of five hand hygiene methods to remove bacterial contamination in a tertiary Vietnamese hospital.

## Methods

Five fingertip imprints of the dominant hand of 134 healthcare workers (HCWs) were sampled to establish the average bacterial count before and after hand hygiene action using: 1) alcohol-based handrub (ABHR); 2) plain soap and water handwashing with filtered and unfiltered water; 3) 4% chlorhexidine gluconate (CHG) hand antisepsis with filtered and unfiltered water.

## Results

Average bacterial contamination of hands before hand hygiene was 1.65 log^10^. *Acinetobacter baumannii*, *Klebsiella pneumonia*, and *Staphylococcus aureus* were the most commonly isolated bacterial pathogens. Highest average count before hand hygiene was recovered from HCWs without direct patient contact (2.10 ± 0.11 log^10^). Bacterial counts were markedly reduced after hand hygiene with ABHR (1.4 log^10^; p<0.0001) and CHG with filtered water (0.8 log^10^; p<0.0001). Use of unfiltered water was associated with non-significant reduction in bacterial counts.

## Conclusion

HCWs carry high levels of bacteria on their dominant hand, even without direct patient contact. ABHR as an additional step may overcome the effect of high bacterial counts in unfiltered water when soap and water handwashing is indicated.

## Disclosure of interest

None declared

